# Corynoxine suppresses lung adenocarcinoma proliferation and metastasis via inhibiting PI3K/AKT pathway and suppressing Cyclooxygenase-2 expression

**DOI:** 10.1186/s41065-024-00343-x

**Published:** 2024-11-07

**Authors:** Liping Chen, Jing Xing, Jiapei Lv, Sainv Si, Huaying Wang, Wanjun Yu

**Affiliations:** 1 Department of Central Laboratory, The Affiliated People’s Hospital of Ningbo University, Ningbo, Zhejiang China; 2 Department of Respiratory, The Affiliated People’s Hospital of Ningbo University, Ningbo, Zhejiang China

**Keywords:** Lung adenocarcinoma, Corynoxine, PI3K/AKT pathway, Cyclooxygenase-2, Proliferation, Metastasis

## Abstract

**Background:**

Lung adenocarcinoma (LUAD) is the most common lung cancer subtype, and the prognosis of affected patients is generally poor. The traditional Chinese medicine *Uncaria rhychophaylla* has been reported to exhibit anti-lung cancer properties. Accordingly, the main bioactive ingredient in *Uncaria rhychophaylla*, Corynoxine, may hold great value as a treatment for lung cancer.

**Methods:**

The impact of Corynoxine on the viability of LUAD cells was assessed using the Cell Counting Kit-8 (CCK-8) assay. Apoptosis in A549 cells was evaluated via flow cytometry. Migration and invasion capabilities were determined through wound healing and Transwell assays, respectively. The key pathways targeted by Corynoxine in LUAD were identified using a network pharmacology approach. Additionally, Western immunoblotting, quantitative real-time PCR (qRT-PCR), and ELISA assays were conducted to validate the underlying mechanisms. The in vivo anti-tumor efficacy of Corynoxine was assessed in xenograft nude mice.

**Results:**

In this study, Corynoxine treatment was found to markedly suppress in vitro LUAD cell proliferative, migratory, and invasive activity. It additionally downregulated Vimentin and promoted E-cadherin upregulation consistent with the disruption of epithelial-mesenchymal transition (EMT) induction while also accelerating apoptotic death. Furthermore, network pharmacology analysis revealed that the PI3K/AKT pathway is a potential target of Corynoxine in LUAD. In vitro assays demonstrated that treatment with Corynoxine resulted in the suppression of PI3K/AKT signaling and a consequent drop in cyclooxygenase-2 (COX-2) expression. These findings were further confirmed in vivo in mice harboring A549 tumor xenografts in which Corynoxine was able to interfere with the PI3K/AKT/COX-2 signaling axis.

**Conclusion:**

This study elucidated the potential effects of Corynoxine in suppressing proliferation and metastasis in LUAD, along with investigating the underlying mechanisms. These data highlight the promise of Corynoxine as a novel therapeutic tool for the treatment of individuals diagnosed with LUAD.

## Introduction

Lung cancer is among the deadliest malignancies at the global level [1], and lung adenocarcinoma (LUAD) is the most common subtype of lung cancer [2]. While there have been major advances in the radiotherapeutic, chemotherapeutic, and targeted treatment of LUAD in recent years, dose-limiting toxicity and therapeutic resistance ultimately contribute to poor outcomes in a large fraction of patients. Accordingly, there remains a pressing need for the development of new drugs that are effective and associated with fewer adverse side effects.

The relationship between cyclooxygenase-2 (COX-2) and oncogenic progression has been an area of active research interest in recent years. COX-2, also referred to as prostaglandin-endoperoxide synthase 2, is responsible for the conversion of free arachidonic acid into prostanoids [3], and its overexpression is a common hallmark of a range of solid tumor types [4]. Indeed, COX-2 overexpression has been reported in lung cancer, with particularly pronounced levels in adenocarcinomas [5]. At a functional level, COX-2 activity can contribute to pro-oncogenic processes such as proliferation, metastasis, and the emergence of anticancer treatment resistance [6, 7]. Efforts to inhibit COX-2 activity or expression thus hold promise as novel treatments for LUAD.

PI3K/AKT signaling is integral to a diverse array of cellular processes including survival and proliferation, with AKT-mediated PI3K pathway activity being essential for lung cancer progression [8]. Multiple reports have shown that PI3K/AKT signaling is required for the expression of the COX-2 gene [9–11], and COX-2 upregulation in response to hepatocyte growth factor (HGF) signaling via this PI3K/AKT pathway has been linked to enhanced invasivity in breast cancer cells [12]. The suppression of PI3K/AKT signaling thus represents an attractive means of robustly suppressing the expression of COX-2 in cancer cells.

*Uncaria rhychophylla* (Miq.) Jacks (Rubiaceae) is widely used in the practice of traditional Chinese medicine and has been reported to exhibit anti-inflammatory, anti-tumor, neuroprotective, analgesic, antihypertensive, and sedative properties [13–15]. Corynoxine is a major bioactive component of *Uncaria rhychophaylla* that has previously been shown to induce autophagic activity and suppress AKT/mTOR signaling in neurons [16]. Corynoxine treatment can also suppress the growth of pancreatic cancer cells through the reactive oxygen species (ROS)-p38 axis [17]. However, no studies to date have evaluated the potential therapeutic effects of Corynoxine when used to treat LUAD.

In the present study, Corynoxine was found to suppress the ability of LUAD cells to proliferate, migrate, and engage in invasivity at least in part by suppressing PI3K/AKT pathway activity and the expression of COX-2, thereby interfering with the progression of this deadly form of cancer.

## Materials and methods

### Reagents and cell lines

Beas-2B, A549, SPC-A1, and NCI-H1299 cells were obtained from Biobw (Beijing, China) and grown in DMEM (Gibco, C11995500BT, USA) containing 10% fetal bovine serum (FBS; Gibco, 10099141, USA) and 100U/ml penicillin/streptomycin (Gibco, 15070063, USA) in a 5% CO_2_ incubator at 37 °C. Corynoxine (> 99% pure, HY-N0901), LY294002 (> 99% pure, HY-10108) and Celecoxib (> 99% pure, HY-10108) were purchased from MedChemExpress (China). In present study, Corynoxine was dissolved in dimethyl sulfoxide (DMSO) as a 100 mM stock solution and stored at − 20 °C. Corynoxine was diluted to obtain the desired concentration in cell culture medium, where the final concentration of DMSO was less than 0.1%. Control cultures received the carrier solvent (0.1% DMSO).

### Cell viability analyses

A cell counting kit-8 (CCK-8; MedChemExpress, HY-K0301, China) assay was used to evaluate the viability of cells. Briefly, Beas-2B, SPC-A1, NCI-H1299, and A549 cells were plated in 96-well plates for 24 h, after which they were treated for an additional 24 h with complete media containing a range of Corynoxine doses (0, 10, 25, 50, 100, 200, or 400 µM). Then, 10 µL of CCK-8 solution was added per well, and absorbance at 450 nm was analyzed following a 2 h incubation.

### Flow cytometry

Following Corynoxine treatment for 24 h, cells were rinsed thrice using PBS, suspended in binding buffer, and stained with FITC and propidium iodide (PI) for 15 min based on provided instructions (KeyGen BioTECH, KGA108, China). A flow cytometer (BD Biosciences, USA) was then used to evaluate cellular apoptosis.

### Wound healing assay

The migration ability was determined by wound healing assay as described previously [18]. A549 cells were cultured to 90% confluence in 6-well plates, after which a 200 µL pipette tip was used for scratch wound generation. Cells were imaged after 0 h and following Corynoxine treatment (0, 25, 50, and 100 µM) for 24 h. The widths of the scratch at 0 and 24 h were observed and photographed using an inverted light microscope.

### Transwell assay

Tanswell assay was used to measure the invasion of A549 cells [19, 20]. Briefly, A549 cells were suspended in media supplemented with Corynoxine (0, 25, 50, or 100 µM) and added to the upper portion of a Transwell insert, while media containing 30% FBS was added to the lower changer. After a 24 h incubation step, invasive cells attached to the lower membrane surface were fixed using methyl alcohol, stained using 0.1% crystal violet, and counted via light microscopy.

### Target Profiling and Interaction Network Construction

Lung adenocarcinoma-specific targets were sourced from GeneCards (https://www.genecards.org). To forecast the potential biological targets of Corynoxine, two publicly accessible databases, TCMSP (https://old.tcmsp-e.com/tcmsp.php) and Swiss Target Prediction (http://www.swisstargetprediction.ch/) were consulted. The chemical structure data of Corynoxine was obtained from the PubChem database (https://pubchem.ncbi.nlm.nih.gov/), and gene symbol standardization was carried out using the UniProt database (https://www.uniprot.org/).

For the construction of the protein-protein interaction (PPI) network, the STRING database version 11.0 (https://string-db.org/) was utilized, specifying *Homo sapiens* as the target organism and setting the interaction score threshold at 0.4. The resultant network was visualized with Cytoscape 3.9.1 (Cytoscape Consortium, Seattle, WA, USA).

### Go and KEGG pathway analysis

After extracting key targets from the PPI network, Gene Ontology (GO) analysis and Kyoto Encyclopedia of Genes and Genomes (KEGG) pathway enrichment were analyzed through the use of DAVID tools. Subsequently, the top 10 GO terms and the top 20 KEGG pathways with a significance level of *p* ≤ 0.01 were chosen for functional annotation clustering.

### Quantitative real-time PCR (qRT-PCR) assay

Total RNA was extracted by the application of TRIzol (Invitrogen, USA). 1 µg total RNA samples were reversely transcribed utilizing the Prime Script™ RT Master Mixture (Takara, Japan). qRT-PCR was performed using SYBR Green (Applied Biosystems, USA). All primers used for qRT-PCR were designed and synthesized by Sangon Biotech (Shanghai, China). The expression was estimated via the 2-^ΔΔCt^ strategy with GAPDH as an internal control. All primers are listed in Table 1.

**Table 1:** Primer sequences for qRT-PCR

Gene name	Forward primer (5’-3’)	Reverse primer (5’-3’)
**PIK3CA**	GGACCCGATGCGGTTAGAG	ATCAAGTGGATGCCCCACAG
**PIK3CB**	TGGAGAGAGAGCAGTTCCAAT	ATCTCTCGGCAGTCTTGTCG
**PIK3CD**	CTTGCCAGGACCTTCCCTCT	GCAGCCATGTCGGTTCTTC
**COX-2**	TTGCATTCTTTGCCCAGCAC	ACCGTAGATGCTCAGGGACT
**GAPDH**	GGAGCGAGATCCCTCCAAAAT	GGCTGTTGTCATACTTCTCATGG

### ELISA assay

After treated with different concentrations of Corynoxine or Celecoxib (40 µM) for 24 h, the prostaglandin E2 (PEG2) were determined in the supernatant by ELISA assay (Elabscience, E-EL-0034c, China) according to the manufacturer’s protocol. The absorbance of the plates was read by a microplate reader. Concentrations of PEG2 were calculated according to the standard curve and presented as pg/mL.

### Western immunoblotting

Cell or tissue sample lysis was achieved with chilled RIPA buffer (Beyotime, P0013B, China) containing phosphatase inhibitors (Sangon Biotech, C500019, China) and PMSF (Solarbio, P0100, China). A BCA kit (Beyotime, P0012, China) was then used to quantify protein content in these samples, after which Western blotting was conducted as in prior reports [21]. Briefly, ~ 30 µg of protein was separated per sample, followed by transfer onto PVDF membranes (Millipore, 0.45 μm, USA). Blots were incubated overnight with primary antibodies prepared in 5% BSA (Solarbio, SW3015, China) at 4 °C, including antibodies specific for Vimentin (1:1000, Abcam, ab20346, USA), Bcl-2 (1:2000, Proteintech, 26593-1-AP, China), Bax (1:200, Santa Cruz, sc-20067, USA), E-cadherin (1:1000, CST, 14472, USA), PI3K p110δ (1:200, Santa Cruz, sc-55589, USA), AKT (1:1000, CST, 4685, USA), p-AKT (1:1000, CST, 4060, USA), and COX-2 (1:1000, CST, 12282, USA), and β-actin (1:5000, TransGen, TC201, China). Blots were subsequently probed with an HRP-conjugated secondary antibody (1:10000; Proteintech, SA00001-1/SA00001-2, China). An Enhanced chemiluminescence solution (Bio-Rad, 1705061, USA) was then used to detect protein bands, which were analyzed using ImageJ.

### Xenograft tumor modeling

All animal model studies described herein received approval from the Experimental Animal Ethics Committee of Ningbo University (Approval number: 10444), and complied with the National Research Council’s Guide for the Care and Use of Laboratory Animals. Male BALB/c nude mice that were 4 weeks of age received a subcutaneous injection of 100 µL PBS containing 2 × 10^6^ A549 cells into the right flank. Tumor volume was measured in each mouse every 3 days with the formula: π/6 × (short diameter)^2^ × (long diameter) [22], with body weight also being measured. When tumor growth at 100mm^3^, mice were randomly assigned to four groups (eight mice per group), and Corynoxine prepared in 0.5% CMC-Na solution of dosage of 30 mg/kg, 20 mg/kg and 10 mg/kg body weight. And the control mice were injected with an equal volume of 0.5% CMC-Na solution. Animals were treated once daily via gavage, and were euthanized following a 21-day treatment period.

### Immunohistochemistry

Tumor sections were prepared at a thickness of 4 μm following paraffin embedding, after which the sections were deparaffinized, rehydrated, and treated with an antigen repair solution. Sections were subsequently blocked for 30 min using 10% normal goat serum, stained overnight with anti-Ki-67 (1:400, Abcam, ab16667, USA) at 4 °C, stained for 1 h with secondary antibody (1:1000, Vectorlab, BA-1000, USA), and the percentage of cells positive for Ki-67 was then quantified following DAB labeling.

### Statistical analysis

Data are reported as means with standard deviations (SD), and experiments were repeated at least three times. Data were compared with Student’s t-tests or one-way ANOVAs with Dunnett’s test for in vitro analyses, whereas in vivo data were compared with two-way repeated-measures ANOVAs with Bonferroni post hoc testing. *P* < 0.05 was considered statistically significant, and GraphPad Prism 7 (GraphPad Software, CA, USA) was used for all statistical testing.

## Results

### Corynoxine suppresses LUAD cell proliferative activity and promotes apoptotic death

**Fig. 1:**
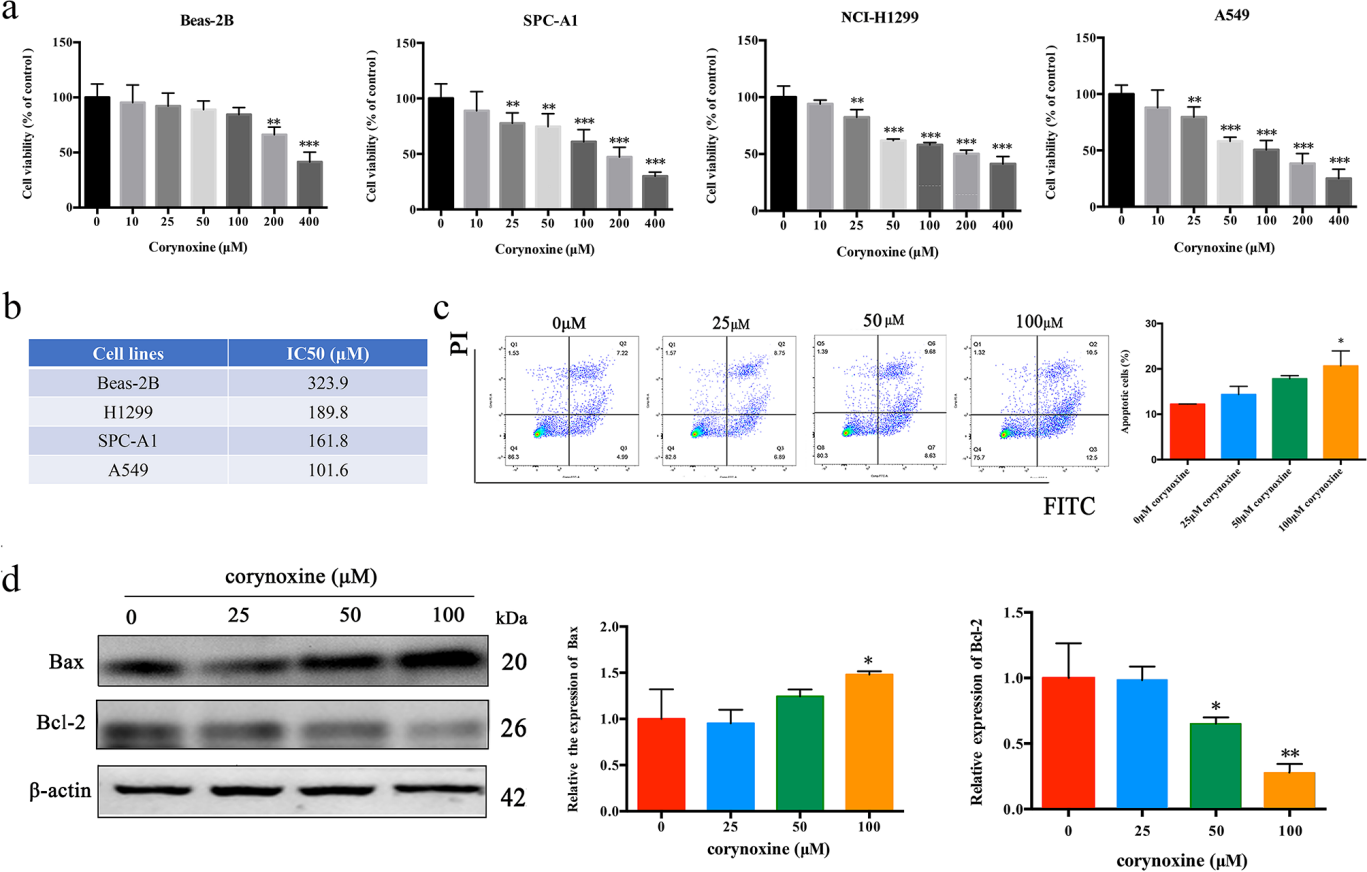
Corynoxine suppresses LUAD cell proliferative activity and induces apoptotic death. (**a**)Viability was evaluated via CCK-8 assay following treatment with a range of Corynoxine doses (0, 10, 25, 50, 100, 200, 400 µM) for 24 h. (**b**) Corynoxine IC50 values for the tested cell lines. (**c**) A549 cell apoptosis was detected by flow cytometry after 24 h treatment with different doses of Corynoxine (0, 25, 50, 100 µM), and the percentage of apoptotic cells was calculated. (**d**) Western immunoblotting was employed to assess Bcl-2 and Bax protein content. Data were expressed as mean ± SD in three independent experiments. *P < 0.05, **P < 0.01, ***P < 0.001

An initial CCK-8 assay revealed the ability of Corynoxine to facilitate the dose-dependent suppression of NCI-H1299, SPC-A1, and A549 cell proliferation, with IC50 values of 189.8 µM, 161.8 µM, and 101.6 µM, respectively (Fig. 1a and b). In contrast, it had little impact on the viability of control Beas-2B human bronchial epithelial cells below a 200 µM concentration (Fig. 1a). Given that A549 cells were the most sensitive to Corynoxine in this preliminary assay, they were used in subsequent experiments. Flow cytometry demonstrated the ability of Corynoxine to promote the apoptotic death of A549 cells (Fig. 1c), with a corresponding rise in the Bax/Bcl-2 ratio in these treated cells (Fig. 1d). Overall, these data suggested that Corynoxine can suppress LUAD cell proliferative activity and induce apoptotic death.

### Corynoxine disrupts A549 cell migratory and invasive activity

**Fig. 2:**
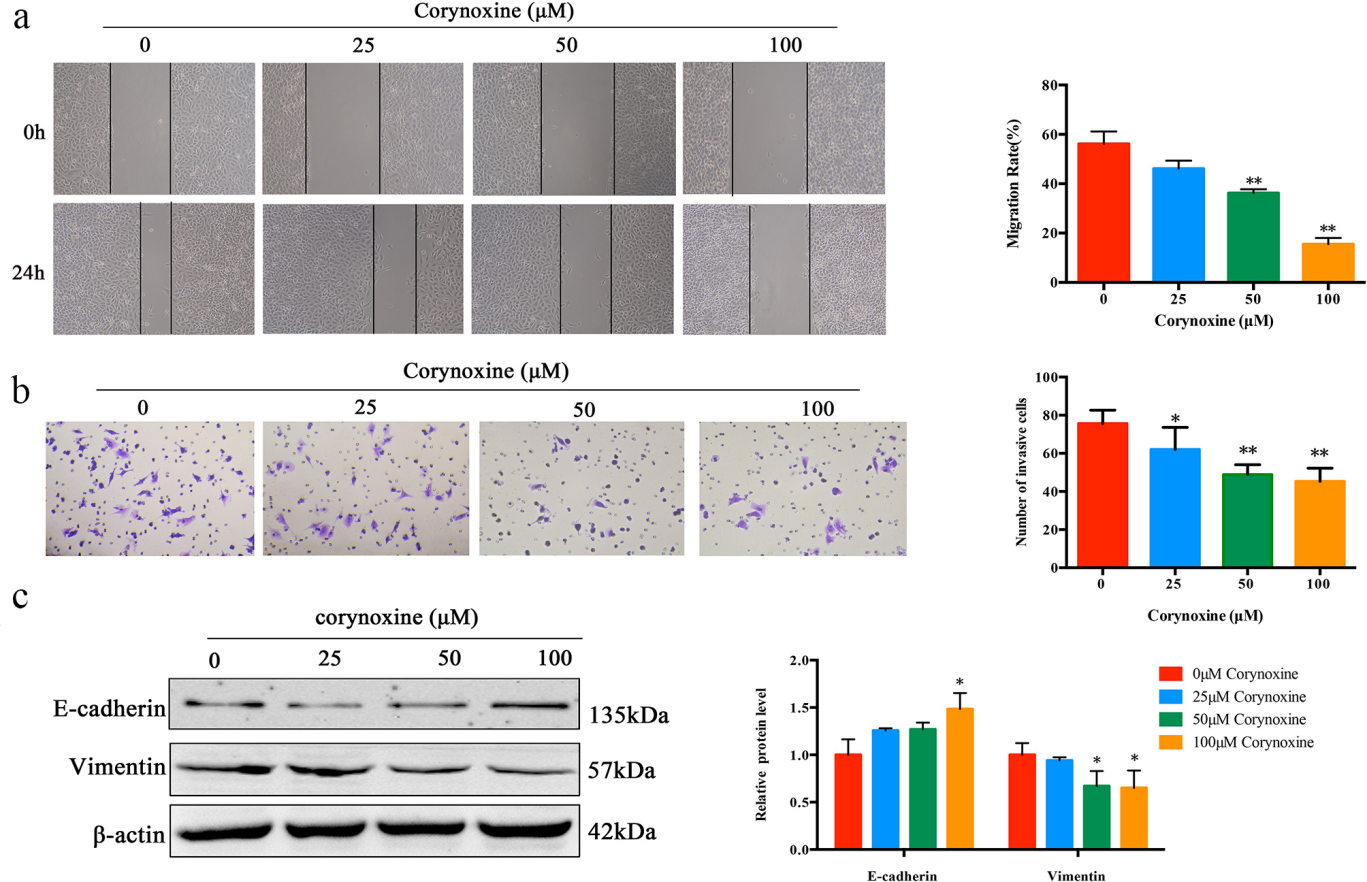
Corynoxine suppresses the invasivity and migration of A549 cells. (**a**) Cellular migration was detected through a wound healing assay. A549 cells were incubated with Corynoxine for 24 h Cell migration was measured by manual counting. Original magnification, 100×. (**b**) Changes in A549 cell invasivity in response to Corynoxine treatment were detected through Transwell assays. Original magnification, 400×. (**c**) E-cadherin, Vimentin, and β-actin were detected via Western immunoblotting. Data were expressed as mean ± SD. *P < 0.05, **P < 0.01

Wound healing and Transwell approaches were next employed to gauge any potential changes in A549 cell invasivity or migratory activity following Corynoxine treatment. These approaches highlighted the ability of Corynoxine to suppress both of these pro-metastatic activities (Fig. 2a and b). Consistently, treatment with a higher dose of Corynoxine (100 µM) resulted in enhanced E-cadherin expression, while a dose-dependent decline in Vimentin expression was also observed in treated cells (Fig. 2c). These results indicated that Corynoxine can suppress the migratory and invasive properties of A549 cells.

### Network pharmacology analysis is conducted on corynoxine-related targets and potential pathways

**Fig. 3:**
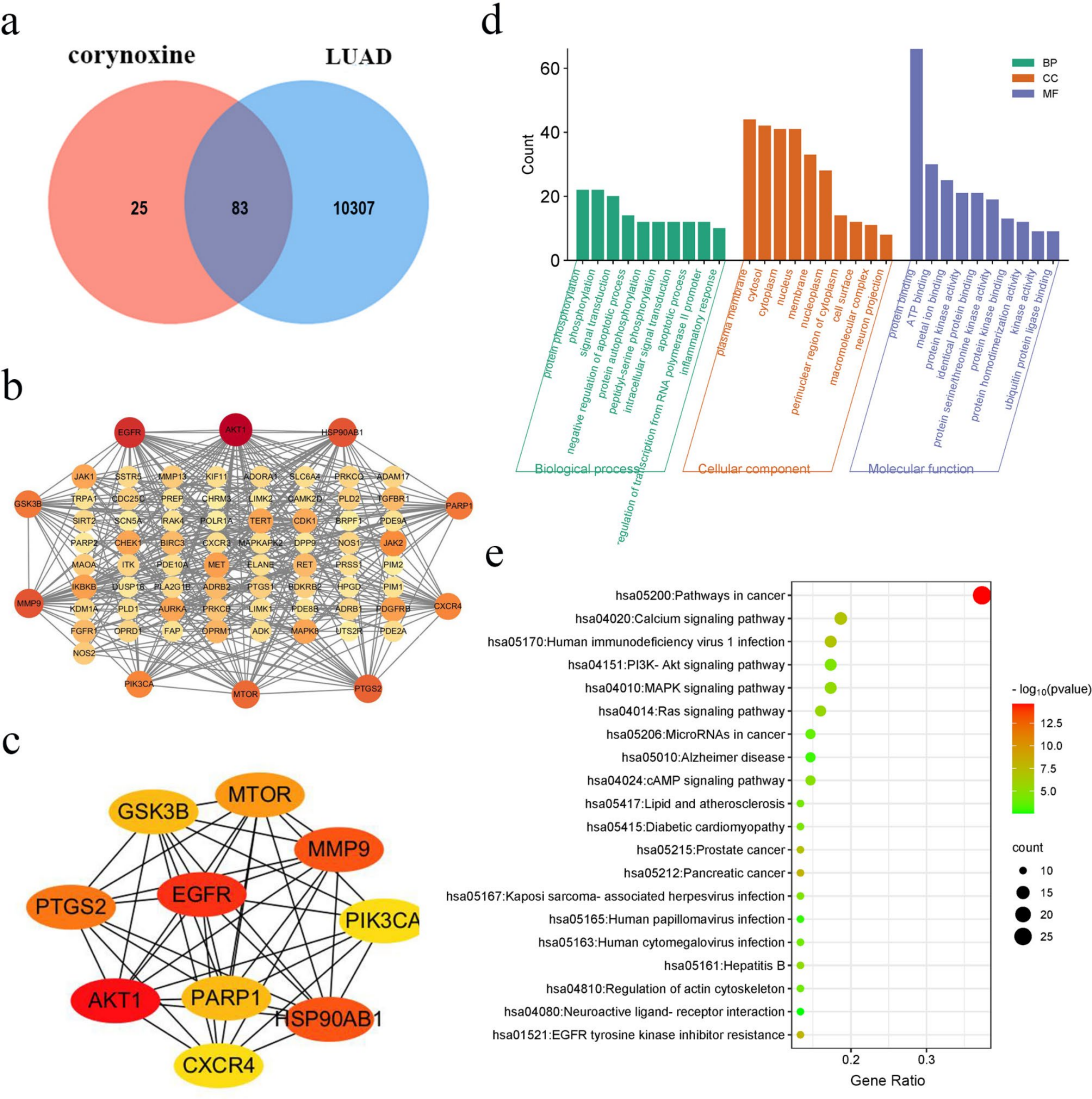
The therapeutic targets of Corynoxine for LUAD were identified through network pharmacology analysis. (**a**) Venn diagram identified the common targets of Corynoxine and LUAD. (**b**) PPI network was constructed. (**c**) The hub network was obtained by screening. (**d**) Top 10 enriched GO terms. (**e**) The top 20 potential pathways were identified through KEGG enrichment analysis

In an endeavor to uncover the targets and pathways modulated by Corynoxine in LUAD, a network pharmacology analysis was conducted. The analysis revealed 108 Corynoxine-associated targets and 10,390 LUAD-related targets, with 83 common targets identified (Fig. 3a). Subsequently, these common targets were utilized in the STRING database to construct a PPI network, comprising 75 targets (Fig. 3b). The top 10 hub genes determined by Cytohubba using the Degree method were AKT1 (degree = 40), EGFR (degree = 33), MMP9 (degree = 28), HSP90AB1 (degree = 28), PTGS2 (degree = 26), MTOR (degree = 25), PARP1 (degree = 23), GSK-3β (degree = 23), CXCR4 (degree = 21) and PIK3CA (degree = 21) (Fig. 3c). Additionally, GO and KEGG analyses were conducted to identify the implicated pathways. The top 10 significantly enriched terms in GO analysis and the top 20 significantly enriched pathways in KEGG analysis are illustrated in Fig. 3d and e, respectively. Notably, cancer pathways were the foremost in KEGG analysis, further supporting Corynoxine’s anti-cancer properties. Furthermore, the PI3K/AKT pathway emerged prominently in the KEGG analysis. Through integrated analysis, it is speculated that the PI3K/AKT signaling pathway may be a primary pathway inhibited by Corynoxine in LUAD.

### Corynoxine inhibits PI3K/AKT signaling pathway to suppress the expression of COX-2

**Fig. 4:**
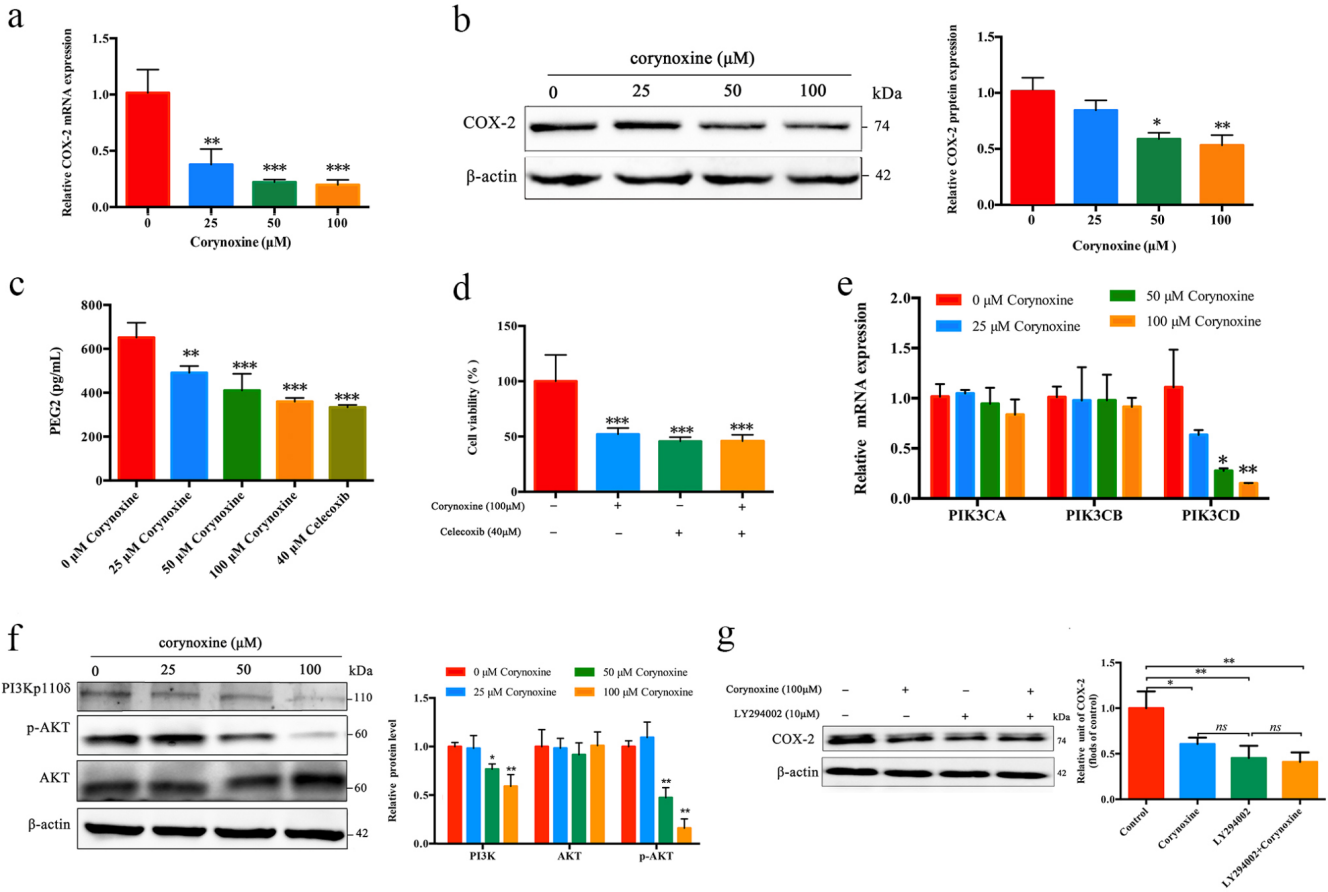
Corynoxine reduces COX-2 levels through PI3K/ATK pathway suppression. After treated with different concentrations of Corynoxine, (**a**) COX-2 mRNA expression was detected by qRT-PCR. (**b**) Western immunoblotting was used to detect COX-2 and β-actin levels. (**c**) After treated with different concentrations of Corynoxine or Celecoxib (40 µM) for 24 h, the level of PEG2 was evaluated by ELISA assay. (**d**) A549 cells were treated with indicated doses of Corynoxine for 24 h after pretreatment with the COX-2 selective inhibitor Celecoxib (40 µM) for 8 h, and the cell viability was determined by CCK-8 assay. After treated with different concentrations of Corynoxine. (**e**) Different subunits of PI3K (PIK3CA, PIK3CB and PIK3CD) were detected by qRT-PCR. (**f**) Western immunoblotting was used to detect PI3K p110δ, p-AKT, AKT, and β-actin levels. (**g**) After treatment with 100 µM Corynoxine or 10 µM LY294002 for 24 h, western immunoblotting was used to assess the expression of COX-2 and β-actin expression. Data were expressed as mean ± SD, *n* = 3. *P < 0.05, **P < 0.01, ***P < 0.001

According to PPI network analysis, it was revealed that PTGS2, also known as COX-2, is the primary target of Corynoxine in treating LUAD. Overexpression of COX-2 has been linked to enhanced proliferative and migratory activities in lung cancer cells [23]. Building upon these observations, levels of COX-2 in A549 cells were examined, revealing a dose-dependent suppression by Corynoxine (Fig. 4a and b). To assess COX-2 activity, the levels of PGE2 were measured in A549 cells exposed to Corynoxine or a 40µM dose of Celecoxib (COX-2-selective inhibitor) for 24 h. Corynoxine demonstrated a significant dose-dependent suppression of PGE2 release into the supernatant of A549 cells (Fig. 4c). Furthermore, to explore Corynoxine’s regulatory impact on COX-2 signaling, A549 cells were pre-treated with Celecoxib (40 µM) for 8 h followed by Corynoxine at 100 µM for 24 h. Notably, the combination treatment did not significantly alter the observed reduction in cell viability compared to individual treatments (Fig. 4d). The KEGG pathway analysis indicated a pronounced enrichment of the PI3K/AKT signaling pathway. Corynoxine was found to inhibit the expression of PIK3CD in a concentration-dependent manner as demonstrated by qRT-PCR (Fig. 4e). Western immunoblotting further confirmed that Corynoxine treatment reduced levels of PI3K p110δ and p-AKT within A549 cells (Fig. 4f). Previous studies have highlighted the role of PI3K/AKT signaling in driving COX-2 upregulation in lung adenocarcinoma cells [24]. To validate this association, A549 cells were treated with the selective PI3K/AKT pathway inhibitor, LY294002. Treatment with LY294002 (10 µM) effectively suppressed COX-2 levels, which were not further reduced by co-treatment with Corynoxine (100 µM) (Fig. 4g). These results reinforce the involvement of the PI3K/AKT signaling pathway in the Corynoxine-induced decrease of COX-2 levels in LUAD cells.

### Corynoxine suppresses tumor growth in mice

**Fig. 5:**
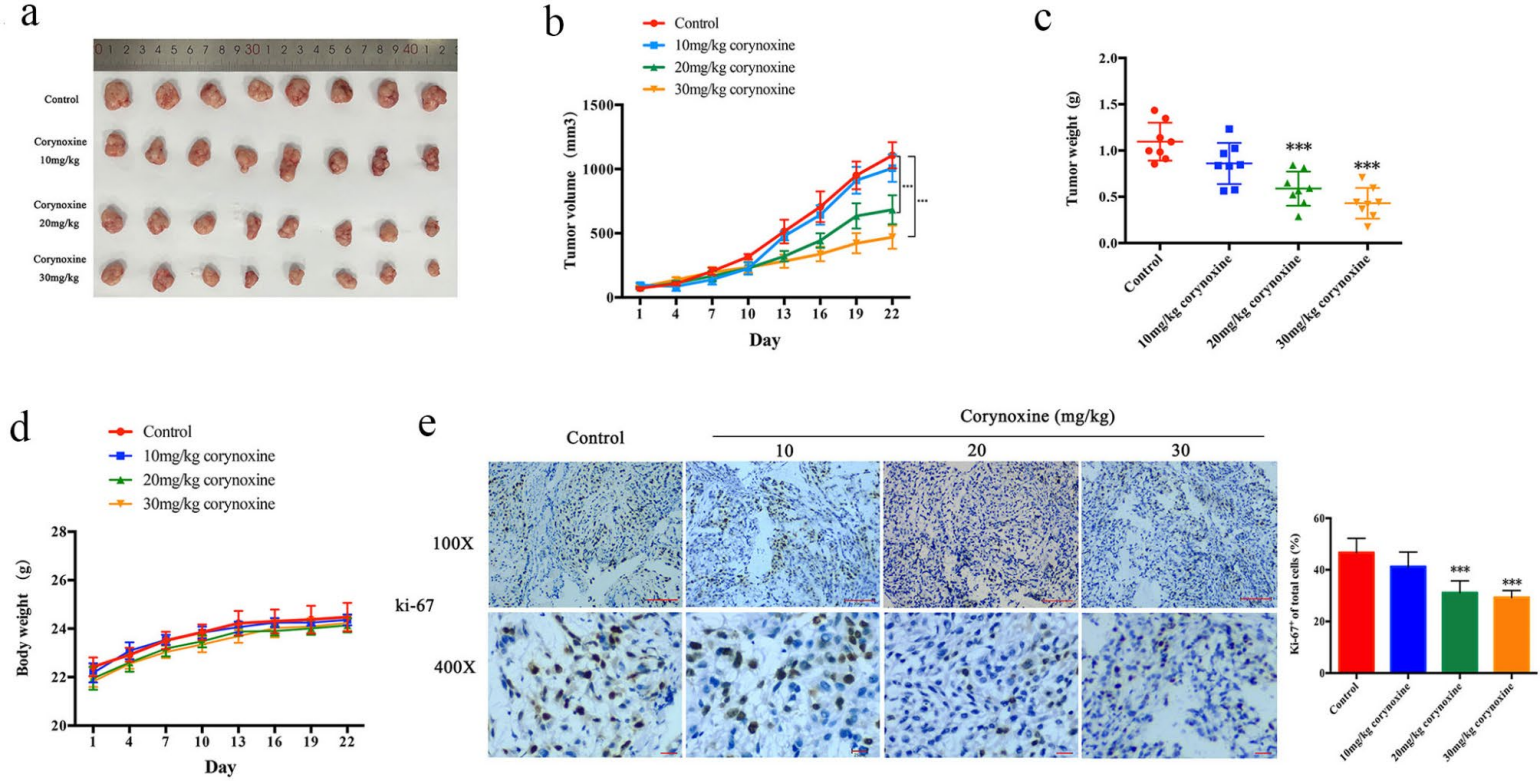
Corynoxine suppresses xenograft tumor growth. (**a**) The tumor pictures, *n* = 8. (**b**) The tumor volumes were measured every 3 days. (**c**) The tumor weight was evaluated at day 22. (**d**) Body weight was assessed every 3 days. (**e**) Ki-67 levels were assessed via IHC staining. Scale bar: 200 μm–50 μm. Data were expressed as mean ± SD. *P < 0.05, **P < 0.01, ***P < 0.001

The impact of Corynoxine on tumor growth was next evaluated with xenograft model mice into which A549 cells had been implanted. As expected, 20 mg/kg and 30 mg/kg Corynoxine dramatically reduced the tumor volume and the tumor weights, compared with the control group (Fig. 5a, b and c). No differences in body weight were observed between these four treatment groups (Fig. 5d), and IHC staining revealed significantly lower Ki-67 levels within the tumors of 20 mg/kg and 30 mg/kg Corynoxine-treated mice (Fig. 5e). Together these findings supported the ability to effectively disrupt tumor growth in vivo without any appreciable toxic side effects.

### Corynoxine suppresses PI3K/AKT/COX-2 pathway activity in vivo

**Fig. 6:**
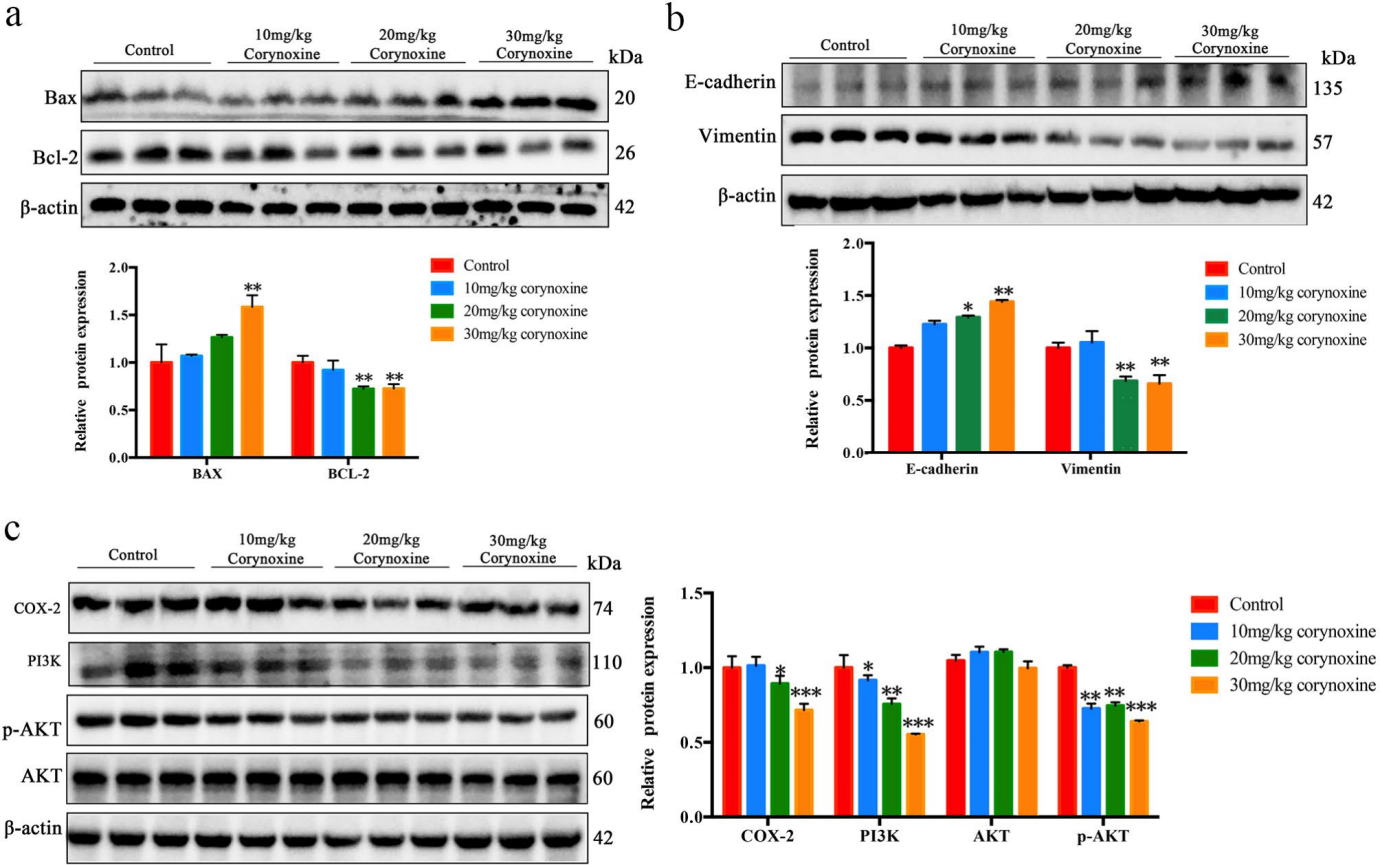
Corynoxine suppresses intratumoral PI3K/AKT/COX-2 pathway activity. (**a**) Bax, Bcl-2, and β-actin protein levels. (**b**) E-cadherin, Vimentin, and β-actin protein levels. (**c**) Intratumoral COX-2, PI3K, AKT, p-AKT, and β-actin levels were detected. Data were expressed as mean ± SD, *n* = 3. *P < 0.05, **P < 0.01, ***P < 0.001

To test the ability of Corynoxine to disrupt oncogenic processes through the PI3K/AKT/COX-2 signaling axis, Bax and Bcl-2 levels were analyzed in the xenograft tumors from these mice, and the results showed that administration of 30 mg/kg Corynoxine up-regulated Bax expression, and 20 mg/kg and 30 mg/kg Corynoxine down-regulated Bcl-2 expression (Fig. 6a). Moreover, E-cadherin was upregulated in 20 mg/kg and 30 mg/kg Coryoxine-treated tumors, whereas Vimentin levels were reducedt, as compared to control mice (Fig. 6b). In addition, COX-2, PI3K, and p-AKT protein levels fell in xenograft tumors from the 20 mg/kg and 30 mg/kg Coryoxine treatment group (Fig. 6c). These findings suggest that Corynoxine can effectively suppress tumorigenic processes through the in vivo inhibition of PI3K/AKT/COX-2 pathway activation.

## Discussion

LUAD remains a highly invasive and deadly form of cancer, with affected patients exhibiting poor 5-year overall survival outcomes [25]. As naturally-derived compounds consist of multiple components that interact with multiple targets. The traditional Chinese medicine *Uncaria rhychophaylla* has been reported to exert antitumor activity, suppressing lung tumor growth [26]. The *Uncaria rhychophaylla* derivative Corynoxine is a 3-substituted indole-2-ketone, and other drugs in this same compound class such as sunitinib malate have been shown to exhibit robust anti-angiogenic and antitumor activities such that it has been applied to treat metastatic renal cell carcinoma and malignant intestinal stromal tumors [27]. Based on the present results, Corynoxine can markedly suppress cell proliferation, invasivity, and metastatic progression while inducing apoptotic death, suppressing COX-2 expression, and inhibiting the signaling via the PI3K/AKT pathway. Interestingly, some Corynoxine analogs, e.g., such as isorhynchophylline was also found to anti-tumor properties[13]. Corynoxine exhibed an IC50 value (A549: 101.6 µM) that was markedly below that reported previously for isorhynchophylline (A549: 236 µM; HepG2: 130 µM), indicating that it may be a more powerful antitumor drug relative to characterized analogs. These factors provide clear justification for the application of coryoxine as a lead compound for the further development of antitumor pharmaceuticals.

PI3K/AKT signaling activity plays a central role in NSCLC progression with the activation of AKT ultimately inhibiting the pro-apoptotic Bcl-2 family proteins Bax and Bad [28]. Corynoxine can suppress cellular proliferation while inducing apoptotic death, and such treatment also led to decreased PI3K and p-AKT/AKT expression in this experimental setting. Moreover, Bax levels rose following Corynoxine administration whereas Bcl-2 levels fell. The proliferation-related nuclear antigen Ki-67 can enable the reliable assessment of tumor cell proliferation [29], and in this study the Ki-67 levels fell in tumors that had undergone Corynoxine treatment.

PI3K/AKT signaling is also vital to the EMT induction process [30]. Tyrosine kinases can induce PI3K activation, thereby leading to PIP3 generation at the plasma membrane that subsequently induces AKT activation through the AKT PH domain. AKT upon activation will phosphorylate several substrates, and such changes may stimulate EMT [31]. EMT induction is closely associated with declining levels of epithelial adhesion marker proteins such as E-cadherin together with the upregulation of vimentin and other mesenchymal factors [32]. In this study, treatment with Corynoxine was sufficient to impair A549 cell migration while promoting E-cadherin upregulation and suppressing Vimentin levels in these cells.

While COX-2 expression levels are minimal in normal tissue, it is commonly upregulated in cancers wherein it can serve to drive proliferative activity and protect against cellular death [33]. In lung adenocarcinoma in particular, COX-2 reportedly facilitates angiogenesis, invasivity, and metastatic progression [34]. The COX-2/PGE2 axis also plays a critical role in EMT induction, which is vital to the augmentation of tumor cell invasion and metastasis for tumors of epithelial origin [35]. Here, treatment with Corynoxine was sufficient to hamper A549 cell invasivity while also downregulating COX-2. Notably, prior work has established COX-2 as a PI3K/AKT signaling target such that interfering with this pathway can reportedly disrupt PGE2-driven COX-2 expression within lung cancer cells [24]. Consistently, treating A549 cells with LY294002 to inhibit PI3K activity was sufficient to reduce COX-2 levels, and co-treatment with Corynoxine did not further decrease such expression, suggesting that the ability of Corynoxine to suppress malignant activities in NSCLC cells is at least partially mediated by the PI3K/AKT/COX-2 axis.

PI3K is a dimer with phosphatidylinositol kinase activity composed of regulatory subunit p85 and catalytic subunit p110 [36]. The current study revealed that Corynoxine effectively suppresses the expression of PIK3CD, which encodes PI3K p110δ, in A549 cells. However, the precise mechanism through which Corynoxine modulates PIK3CD mRNA expression in A549 cells remains unclear. MicroRNAs (miRNAs) are noncoding RNAs that play a crucial role in post-transcriptional gene regulation, influencing various biological processes such as cell proliferation, division, migration, and apoptosis [37, 38]. These miRNAs exert their inhibitory effects on gene expression by targeting the 3′-untranslated region (UTR) of mRNA, leading to mRNA degradation and translational repression[38]. Previously, some Corynoxine analogs, e.g., such as isorhynchophylline can modulate the expression levels of miRNAs [39, 40]. Based on these observations, it is hypothesized that the potential mechanism by which Corynoxine inhibits PI3K expression may involve the modulation of miRNAs to trigger the degradation of PIK3CD mRNA.

## Conclusion

In conclusion, the present results offer novel evidence for the ability of Corynoxine to suppress the development and progression of LUAD tumors at least in part by targeting the PI3K/AKT/COX-2 pathway. These data support the potential value of further developing Corynoxine-based regimens for the treatment of individuals suffering from LUAD.

## Data Availability

No datasets were generated or analysed during the current study.
